# Sir William Osler, Oxford and "The Open Arms"

**Published:** 1992-08

**Authors:** 

**Affiliations:** Detchant Former President, General Medical Council, British Medical Associaion and Royal Society of Medicine (UK); former Warden, Green College, Oxford


### Sir William Osler, Oxford and 'The Open Arms'*

**Lord Walton of Detchant**
Former President, General Medical Council, British Medical Associaion and Royal Society of Medicine (UK);
former Warden, Green College, Oxford

* A lecture delivered to the Bristol Branch of the.BMA on 22 January 1992, based upon one delivered to the Japanese Osler Society in Tokyo in August 1990 and subsequently published by that Society in Japanese.

### INTRODUCTION

Without question, Sir William Osler was one of the greatest physicians who ever lived. The existence of 19 Osler Societies throughout the world, including one in Japan, which have been established to hallow his memory and to encourage the study of the history of medicine and the other subjects which were close to his heart must testify to that fact. It was the 14 years that he spent in Oxford as the Regius Professor of Medicine (regius professors are appointed by the sovereign) which finally established and greatly enhanced his world-wide reputation.

William Osler was born in 1849 in Bond Head, a small village some 30 km. north of Toronto, where his father was a priest in the Anglican church. In 1857 Canon Osler, as he then was, was given the rectorship of Dundas, just outside the city of Hamilton, Ontario, where William Osler attended the grammar school. There he proved to be a holy terror, playing every conceivable prank on his teachers. Once he and his schoolmates locked a flock of geese in the public school-room. On another occasion they unscrewed the desks and benches from the floor and hid them in the attic. Eventually he and his friends were expelled from school and in 1864 he was then sent to a private school in Barry, close to Toronto. Despite his propensity towards practical joking, he proved to be an outstanding pupil and his father, who had originally emigrated to Canada from Cornwall, proved to have a life-long influence upon William's taste for good literature. As a boy he read widely and early in life began one of his life-long habits of writing down references or quotations in a notebook of any items in books which took his interest. The first book he ever bought was the Globe Shakespeare, which was later stolen, but his second was the 1862 edition of Sir Thomas Browne's "Religio Medici", a book which remained his life-long favourite and from which he quoted on every possible occasion. This was the start of his fine library. He was always a bibliomaniac and could never resist a literary masterpiece, especially in latter years one with a bearing upon medicine. Indeed when he was resident in Oxford he often crept into the house quietly without warning his wife of his presence in order to be able to secrete away in his library his latest literary purchase. Before he died he had collected in his library every one of the 52 editions of "Religio Medici", and just before his death he completed a catalogue of certain incunabula (medical books printed up to 1480), many of which he had in his own library which after his death formed the core of the splendid Osler Library in McGill University in Montreal.

After completing his school career, Osler became a medical student at McGill University, Montreal, graduating with distinction, though it is fair to say that few of his contemporaries saw in him the seeds of greatness which were subsequently to become so evident in his glittering career. It is nevertheless notable that his first publication, written when-he was 18 yearsof age, appeared in the British publication "Science Gossip" on 1 February 1869 and was based on microscopic studies of algae in water collected in a marshy inlet of Lake Ontario at Dundas.

This demonstrated early evidence of the active, original and enquiring mind which so dominated his subsequent career, coupled with the compassion and humanism of an outstanding caring physician.

Soon after graduation in medicine, Osler decided to go to Britain for a period of postgraduate study. Canon Osler and the family collected sufficient funds to allow him to do so; he had been much impressed in his student career by R. Palmer Howard, the Professor of Medicine at McGill from 1870 to 1872, and it was Howard's ambition that William should train in ophthalmology with the ultimate objective of joining the teaching staff of McGill and of Montreal General Hospital. He therefore went to London in September 1872 and worked in the physiology laboratory of University College with John Burdon Sanderson on the advice of William Bowman, England's best-known and most talented ophthalmologist. While working in physiology, he received the disturbing news that a more senior candidate for the post of ophthalmologist in Montreal, Frank Buller, had emerged and was an applicant for the house surgeon appointment at Moorfields upon which young William had set his heart. In the event, as Buller was appointed, Osler decided not to train in ophthalmology but, following in Howard's footsteps, became determined to cultivate the whole field of medicine as it had never been cultivated before.

On his return to McGill in July 1874, he was appointed to the post of Lecturer upon the Institutes of Medicine at the age of 25. The impression which he made upon his class can be gleaned from the remarks of a student who attended his first lecture:

> "The lecture began with an explanation of the old Edinburgh term of Institutes of Medicine. Then in bold outline he sketched inorganic and organic matter, vegetable and organic life, vital force, and closed with a description of cellular life and an outline of future lectures. From that hour, physiology was an attractive study and the lectures like unto the Gods."

In April 1875 Osler was officially appointed professor and demonstrated prodigious industry. He worked first of all in pathology, gave courses for students in the autopsy theatre and prepared a fine collection of pathological specimens in the McGill Museum and several bound volumes of pathological reports. This no doubt was what led him, when he opened the new Pathology Institute at the Royal Infirmary in Glasgow in 1911, to describe the function of a Pathology department within a hospital as "the place where the teaching is done, where ideas are nurtured, where men dream dreams and thoughts materialise into researches upon the one great problem that confronts the profession in each generation - the nature of disease". His appointment as pathologist at McGill was quickly followed by a further appointment as physician to the Montreal General Hospital where he brought his students on the wards face to face with clinical problems which he encouraged them to solve for themselves. Formerly their knowledge of disease had been gathered from lectures and from seeing patients in front of a class in an amphitheatre. When he went to Philadelphia in 1884 as Professor of Medicine, he once again found that bedside instruction was almost nonexistent. In the university hospital he spent the greater part of the morning in the wards with an increasingly enthusiastic group of students around him, and in the entrance to the present university hospital in Philadelphia the famous picture of Osler teaching in the grounds of the hospital at the bedside is seen by every visitor.

In 1889 he made yet another move to the newly-established Johns Hopkins Hospital in Baltimore where, as Foundation Professor of Medicine, with his co-professors Welch Pathology), Kelly (Gynaecology) and Halstead (Surgery) he continued his work and before long these four men, with the help of a series of brilliant assistants, made this hospital one of the finest and best organised in America. Yet again he organised his clinical, laboratory and ward teaching to a degree never before known in America and students at last were admitted to positions of real responsibility on the wards. Many of them were prevailed upon to publish research work that they had done in the clinical laboratory. And while Osler himself made no single fundamental discovery based upon scientific research, he nevertheless published some 730 papers on subjects related to medicine in journals both well-known and obscure, in all parts of Britain and North America. He also published his notable textbook "Principles and Practice of Medicine" which made his name known to every medical student the world over until virtually the beginning of the Second World War.

It is, of course, easy to pick out from Osler's writings many aphorisms as valid today as they were in his time. Neurologists like myself might perhaps question his comment that "Probability is the rule of life, especially under the skin. Never make a positive diagnosis". But in these days of AIDS and of the growing problem of alcoholism in our society, one can only reflect upon his apposite dictum: "Who serves the gods dies young - Venus, Bacchus and Vulcan send in no bills in the seventh decade". He repeated similar advice in a letter on "Rules for a sensible young fellow", written to A.L. Smith on 20 March 1907, recently resurrected by Giles Bullard in the 1986 Balliol Record and brought to my attention by Nicholas Dewey. His comment to the effect that "Common sense nerve fibres are seldom medullated before forty - they are never even seen with the microscope before twenty" should perhaps be assessed along with the saying of Oliver Wendell Holmes the elder, whom he much admired, who wrote that "Science is a first-rate piece of furniture for a man's upper storey if he has common sense on the ground floor". I always enjoyed his wry comment on one of the medical fashions of his age when he said that "the mental kidney, more often than the abdominal, is the one that floats". And how can one forget that "although one swallow does not make a summer, one tophus makes gout and one crescent malaria".

### OSLER'S DEPARTURE FROM AMERICA

In 1894 Osler attended a meeting of the British Association for the Advancement of Science in Oxford and at that time was honoured by the University by being awarded an honorary Doctorate of Science honoris causa. During that meeting he took lunch with Sir Henry Acland and saw in his library a triptych of paintings of Linacre, Sydenham and Harvey which so impressed him that he had two copies made, one of which went to the Medical School at the University of Pennsylvania, where it still hangs, and the other he took with him to his home in Baltimore; later, when he moved to Oxford, the copy of the triptych was installed above the fireplace in his study at 13 Norham Gardens where, thanks to the courtesy of Dr. Alastair Robb-Smith, a further copy has now been installed. At that time it is clear that he had no wish to leave Baltimore where he proved to be enormously successful not only as a teacher and clinical researcher but also as a fund-raiser. He was also in continual demand as a consultant and received many attractive offers from other medical schools, being offered Chairs of Medicine at Jefferson Medical College, Philadelphia, and at Harvard in 1891, and in 1892 McGill tried to tempt him back to fill the chair once held by his mentor, Palmer Howard. During the 16 years he spent in Baltimore he married Grace Revere, had a son (Revere) and gained increasing fame through his writings, his active involvement in medical societies and his popularity as a consultant, but despite enormous energy, a keen sense of organisation and the ability to put odd moments to good use (Key 1989) Osler grew tired of the demands made in response to his growing fame. In his Presidential Address to the American Osler Society, Dr. W.B. Fye mentioned two revealing quotes, in one of which Osler told William Sidney Thayer, "I am on the downgrade, the pace of the last three winters has been such that I knew I was riding for a fall. Better to get out decently in time, and leave while there is still a little elasticity in the rubber.". He then said to Weir Mitchell, who had written extensively on the emotional dangers of overwork, "I am tired of the incessant racket of my present life. I tried hard last winter to cut off some of the work but without any success, and I am often on the edge of a breakdown.". And it is clear that Oxford had long appealed to Osler as a place to work. At the time of the British Association meeting he said "I have lost my heart to Oxford and I feel like the Queen of Sheba after Solomon had displayed all the treasures of his house". Grace Osler, too, was becoming increasingly concerned about the enormous pressures to which her husband was subjected in his life in the United States and hence when in 1904 his former teacher, Sir John Burdon Sanderson, asked if he would consider the possibility of accepting the Regius Professorship of Medicine at Oxford which he (Sir John) had just vacated, the offer came as something of a godsend and, with the full support of his wife, he confirmed that he would be interested and subsequently learned from the Prime-Minister, Mr. Balfour, that the King wished to appoint him to that chair. He took up the appointment on 27 May 1905 ant held it until 1919. Oxford at that time had no clinical school and all of its medical students were preclinical undergradates. As soon as he arrived he started to take part in the introductory clinical course for these students organised by the Radcliffe Infirmary staff and gave masterly demonstrations of the technique of physical examination. At 10 o'clock sharp each Sunday morning he conducted an informal teaching ward round and every Tuesday afternoon gave clinical demonstrations for the local general practitioners and postgraduates. He also embarked upon a series of lectures and demonstrations in the history of medicine and of science, but above all he enjoyed informal meetings with students. Having purchased 13 Norham Gardens, a very substantial Victorian house close to the Radcliffe Infirmary, he would frequently invite students and staff and visitors to his home for tea or supper, would then take them to the library to look at and discuss books, their authors and their significance, and in this way inspired many young men and women to read and discover for themselves. So many and varied were the guests at his house that it came to be called "The Open Arms". Students, teachers, refugees, soldiers, anyone with the vaguest connections, dropped in there and was always welcome. On the day of a conference it would be no new thing for him to bring 120 physicians to tea, having in the morning as he went out hinted that he would bring in "about a dozen" at tea-time. In Oxford he was a friend of young and old alike and especially with children. Often on visiting a home for the first time he would sneak off to the nursery and play for hours with the children, who worshipped their "Doccy 0", as they called him. One particular consultation with the desperately ill son of a physician in Oxford is well remembered. The child was suffering from severe whooping cough and Osler called wearing his bright red Oxford D.M. robes, on the way to an official University function. After examining the child, he gave the parents a somewhat gloomy prognosis which was belied by the fact that soon after the consultation the child began rapidly to recover. He then insisted that the nice doctor should call to see him again wearing his regalia, a request with which Osler willingly complied.

Osler's interest in the history of medicine was not purely academic. He played the leading part in fusing the many London medical societies into the one Royal Society of Medicine in the early part of this century, and in Philadelphia, Baltimore and Oxford he organised bibliographical societies and awoke sleeping librarians into a new life of activity and reorganisation. As a Curator of the Bodleian Library in Oxford, he played a great part in the extensive reorganisation and remodelling of the library just before the First World War.

### OSLER THE TEACHER

What a wonderful teacher Osler must have been. When one reads again his essay on "Teacher and student", in which he stressed the importance of acquiring a significant core of knowledge, based on the appreciation of principles rather than on the cramming of fact, he emphasised system and method, pointing out a lesson which all too many medical students found difficult namely the importance of an orderly arrangement of one's work and the organisation of one's time. While the practice of medicine is surely an art, it must be firmly based upon science/ and scientific reasoning. Having helped to write the General Medical Council's (GMC) Recommendations on Basic Medical Education in 1980, I was surprised to turn back to Osler and to find that some of what I had imagined might be personal gems were not new. In relation to 'Thoroughness' he confirmed that it was necessary to acquire a deep acquaintance of chemistry, anatomy and physiology and of the great principles based upon them. Students must also become familiar with methods by which knowledge is advanced (i.e. research) and must acquire such a knowledge of diseases and of life emergencies and of the means for their alleviation that they may become safe and. trustworthy guides for their fellow men. Only through such a systematic approach did he believe that charlatanism could be avoided. But he also roundly condemned cocksureness;sureness of opinion leading to conceit and stressed the vital importance of humility. I share his admiration for Cowper, who said: "Knowledge and wisdom, far from being one, have oft times no connection. Knowledge dwells in heads replete with thoughts of other men, wisdom in minds attentive to their own. Knowledge is proud that he has learned so much, wisdom is humble that he knows no more." Osler saw as fundamental that master word in medicine which he suggested looms large in meaning. He regarded it, and I quote, as "the open sesame to every portal , the great equaliser in the world, the true philosopher's stone"; he thought that it would make the stupid man bright, the bright man brilliant and the brilliant student steady. "With it all things are possible and without it all study is vanity and vexation. To the youth it brings hope, to the middle-aged confidence, to the aged repose. It has been directly responsible for all advances in medicine during the past 25 centuries. Laying hold upon it, Hippocrates made observation and science warp and woof of our art. Galen so read its meaning that 15 centuries stopped thinking and slept until awakened by Vesalius, the very incarnation of the master word. Harvey gave impulse with its inspiration to a larger circulation than he recognised, Hunter sounded all its heights and depths and stands out in our history as one of the great examples of its virtue. With it, Virchow smote the rock and the waters of progress gushed out, while in the hands of Pasteur it proved a very talisman to open to us a new heaven in medicine and a new earth in surgery." The master word, of course, was 'work'.

### OSLER THE PHILOSOPHER

Not only was Osler a great physician and teacher, but clearly he loved his profession and served it to the end of his days. He regarded the guild of doctors as being of noble ancestry, demonstrating solidarity without insularity, and a progressive character allowing the advance of knowledge and developments in medical science and in patient care, coupled with the all-pervasive benevolence and human understanding so fundamental to its practice. Among the sins which he saw in some colleagues were those of excessive nationalism, provincialism and parochialism. When I re-read his comments on "Chauvinism in Medicine" I recalled the invitation I received in 1987 from the editor of the Oxford Medical School Gazette inviting me to comment upon the seven deadly sins as applied to medicine. I was prompted to consider how these sins, namely lust, intemperance, ire, avarice, sloth, pride and envy, could be interpreted in a medical setting; I concluded that all might at times be relevant, even if their respective importance in relation to medical practice may differ from the way that each is regarded by society as a whole.

Speaking from my experience as past President of the GMC, if one believed the sensational commentaries of the tabloid press upon the proceedings of its Conduct Committee, some doctors, driven by lust, seem to indulge in remarkable sexual activities with patients of the opposite sex. But the proportion of cases involving sexual misdemeanour is small (3 out of the 53 cases handled by the committee in 1988) - a tiny problem when compared with the 24 doctors accused of serious neglect or disregard o£ their professional responsibilities.

**Intemperance** is a syndrome occasionally seen in doctors and we cannot be proud of the fact that until about 1980 the incidence of cirrhosis of the liver in the UK was more than three times as great in doctors as in the general population. Its incidence in members of the profession is now falling, but nevertheless addiction to alcohol or drugs is still the commonest cause of reference to the GMC's health procedures. Between 1980 and 1988 over 150 doctors were placed under supervison or had their registration suspended for this cause and about 10 doctors a month have regularly been referred to the National Counselling and Welfare Service for sick doctors.

Also worrying is **ire**. The more I have seen of medicine in 45 years of clinical practice, the more I have recognised the crucial need for good communication between doctors and patients and the necessity of compassionate and humane management of sick people even when, the doctor is weary and even when provoked or irritated by inconsiderate or irrational behaviour (patients, like doctors, sometimes lose their tempers and behave badly). But times out of number I used to receive letters at the GMC from patients suggesting that doctors had been rude, clumsy, arrogant, thoughtless or just simply unsympathetic, unfeeling or unkind. They said; "I don't want the doctor 'struck off'; please write and ask him (or her) to be nice to people in future" — but that, of course, the Council couldn't do without invoking the full panoply of quasi-legal procedures. **Avarice**, too, falls outside the GMC's statutory powers even though some letters complained about what, prima facie, looked like gross overcharging in private practice. I hope that the profession itself, with appropriate local procedures, can satisfy public concern about such matters.

**Sloth**, if I interpret the word literally, is I think rare among doctors in hospital practice who are all too often, whether in junior resident posts or even when more senior, overworked and weary. And in family practice, the third night call during an epidemic or the stress of coping with an importuning patient in a crowded office can impose almost intolerable strains on a doctor's reserves of patience and understanding. But sick people are often frightened people; the diagnosis of meningitis or of a perforated appendix in the middle of the night, followed by prompt action, can excuse several unnecessary calls which only an expert could judge with hindsight to have been unnecessary. No, sloth is not usually a problem with doctors - but perhaps the most difficult lesson of all for the young doctor to learn (as I said earlier) is how, in the face of competing demands and responsibilities, to be able to organise his or her time.

And so we are left with **pride** and **envy**. Relevant to medicine? Of course they are. If (with apologies to Rudyard Kipling) pride leads you to rely upon your own diagnostic skill and to be so self-confident as to refuse the legitimate wish of a patient to seek a second opinion, it comes before a fall. If intellectual and professional arrogance cloaks your opinions with an aura (to yourself) of infallibility, if that arrogance leads in turn to an inability or unwillingness to talk to a patient on equal terms, if you are unable or unwilling to admit, on occasion, your uncertainties or even your mistakes, and if pride or envy leads you unjustifiably to criticise or disparage the views or advice given by a professional colleague, these are indeed deadly medical sins. The days of 'doctor's orders' are long past; the doctor/patient relationship is a partnership in which-the doctor offers advice but it is up to the patient to decide whether or not to accept it.

**EPILOGUE**

May I finally comment upon some of the fashionable but false antitheses which were so effectively demolished by Sir Douglas Black in his Rock Carling Lecture for 1984? Of his many verbal, philosophical and scientific gems I can only select a few. When Faraday was asked by one sceptical of the value of science "What use is electromagnetism?", his response was brief but compelling: "What use is a baby?" he said. It is not what it is, but what it becomes; today's basic science discovery is tomorrow's advance in patient care. All of us in clinical medicine know that there is no antithesis between the scientific and so-called holistic methods or between the scientific and the compassionate and caring; all doctors surely strive to practise whole-patient medicine, and the good doctor is just as concerned with disease prevention as with cure, Nevertheless, Black did a valuable service in stressing that those who feel that business skills may be used to buy results in the ever-changing and ever-challenging field of medical research must recognise that 'lavish finance can be impotent in the face of unripe time'. Research which does not define precisely a question to be asked, methods of answering it and a route to be followed may be as fruitless in medicine as is that which goes astray because of deficiencies in clinical knowledge, skills or experience.

As Osler so clearly indicated many years ago, clinical and laboratory science, combined with communication skills, must continue to be partners in our aim of serving as best we can our patients, future patients, knowledge itself and society. These may be truisms but it is upon their full recognition and application in our everyday contact with patients and in the Osler tradition that good clinical practice depends.

**REFERENCES**

BLACK, SIR DOUGLAS. (1984). An anthology of false antitheses. Rock Carling Lecture. *Nuffield Provincial Hospital Trust, London.*

BUCHANAN, W.W. (1990). Sir William Osler of Dundas. *Journal of the Royal Society of Medicine,* **83**, 45.

FYE, W.B. (1989). William Osler's departure from North America; the price of success. *The New England Journal of Medicine,* **320**, 1425.

NUFFIELDWORKING PARTY ON COMMUNICATIONS. (1980). Talking with patients: a teaching approach. *Nuffield Provincial Hospitals Trust, London.*

NUFFIELD WORKING PARTY ON COMMUNICATIONS. (1984). Doctor to doctor: writing and talking about patients. *Nuffield Provincial Hospitals Trust, London.*

NUFFIELD WORKING PARTY ON COMMUNICATIONS. (1986). Partnership or prejudice: communication between doctors and those in the other caring professions. *Nuffield Provincial Hospitals Trust, London.*

ROBB-SMITH, A.H.T. (1989). The story behind the Osler plaque. *Amended from Oxford Medical School Gazette*, **28(3), 18; 29(1), 17; 29(2), 18**.

WALTON, LORD. (1989). The 'Open Arms' reviving: can we rekindle the Osler flame? *American Osler Society, Inc., USA.*

